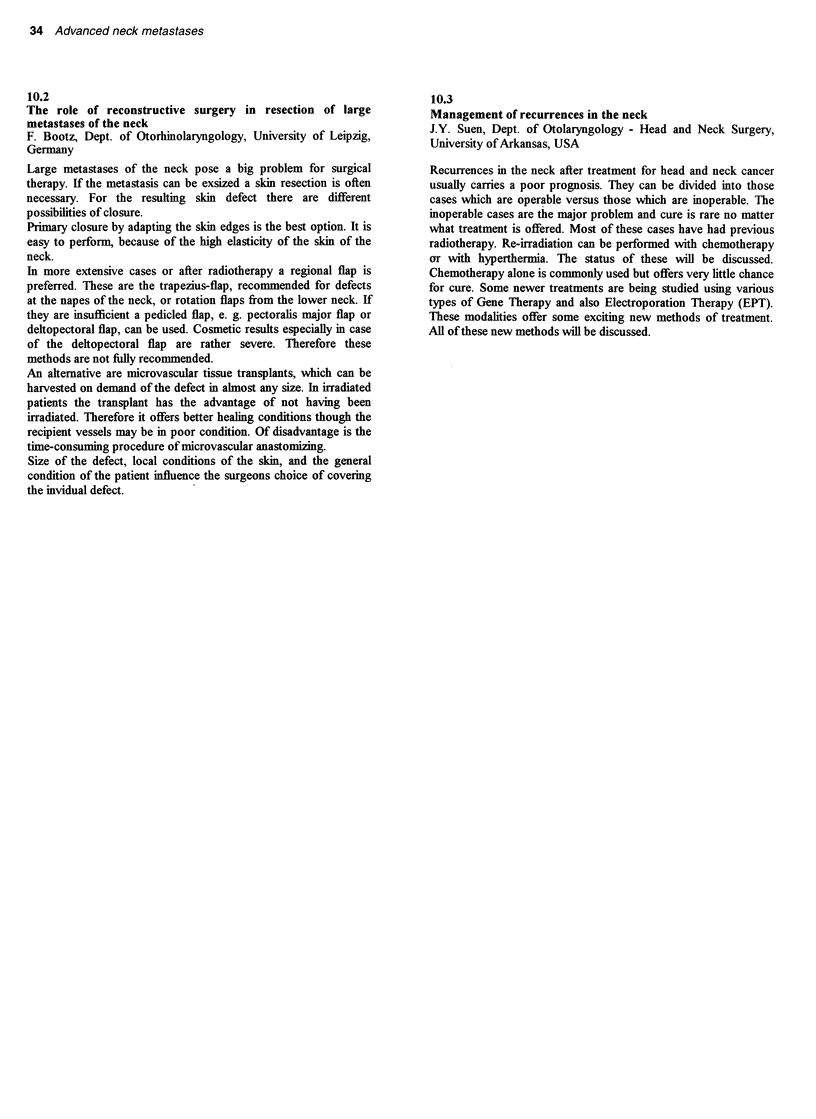# Advanced neck metastases, Recurrences

**Published:** 1998

**Authors:** 


### 10.2
### The role of reconstructive surgery in resection of large metastases of the neck

F. Bootz, Dept. of Otorhinolaryngology, University of Leipzig, Germany

Large metastases of the neck pose a big problem for surgical therapy. If the metastasis can be exsized a skin resection is often necessary. For the resulting skin defect there are different possibilities of closure.

Primary closure by adapting the skin edges is the best option. It is easy to perform, because of the high elasticity of the skin of the neck.

In more extensive cases or after radiotherapy a regional flap is preferred. These are the trapezius-flap, recommended for defects at the napes of the neck, or rotation flaps from the lower neck. If they are insufficient a pedicled flap, e. g. pectoralis major flap or deltopectoral flap, can be used. Cosmetic results especially in case of the deltopectoral flap are rather severe. Therefore these methods are not fully recommended.

An alternative are microvascular tissue transplants, which can be harvested on demand of the defect in almost any size. In irradiated patients the transplant has the advantage of not having been irradiated. Therefore it offers better healing conditions though the recipient vessels may be in poor condition. Of disadvantage is the time-consuming procedure of microvascular anastomizing.

Size of the defect, local conditions of the skin, and the general condition of the patient influence the surgeons choice of covering the invidual defect.

### 10.3
### Management of recurrences in the neck

J.Y. Suen, Dept. of Otolaryngology - Head and Neck Surgery, University of Arkansas, USA

Recurrences in the neck after treatment for head and neck cancer usually carries a poor prognosis. They can be divided into those cases which are operable versus those which are inoperable. The inoperable cases are the major problem and cure is rare no matter what treatment is offered. Most of these cases have had previous radiotherapy. Re-irradiation can be performed with chemotherapy or with hyperthermia. The status of these will be discussed. Chemotherapy alone is commonly used but offers very little chance for cure. Some newer treatments are being studied using various types of Gene Therapy and also Electroporation Therapy (EPT). These modalities offer some exciting new methods of treatment. All of these new methods will be discussed.